# Typical presentation of pulmonary lepidic adenocarcinoma: a rare case report

**DOI:** 10.11604/pamj.2020.36.11.22660

**Published:** 2020-05-11

**Authors:** Rajae Azzeddine, Ismail Abderahmani Rhorfi, Adil Zegmout, Hicham Souhi, Hanane Elouazzani, Ahmed Abid, Hafsa Chahdi, Mohamed Tbouda

**Affiliations:** 1 Department of Pneumology, Military Hospital Mohamed V, Rabat, Morocco; 2 Department of Pathology, Military Hospital Mohamed V, Rabat, Morocco

**Keywords:** Lung adenocarcinoma, alveolar opacity, pathology

## Abstract

Bronchioloalveolar carcinoma (BAC) is a rare subtype of adenocarcinoma of lung with distinct features and distinctive characteristics. It accounts approximately for 4% of lung cancers. In the following study we report a rare observation of a 50 years old female with a clinical, radiological and histological presentation, which is typical of an invasive mucinous lepidic adenocarcinoma formerly named BAC.

## Introduction

Bronchioloalveolar carcinoma (BAC) accounts for approximately 4% of lung cancers [1], it is an uncommon subset of lung adenocarcinoma that develops from terminal bronchiolar and acinar epithelia of the lung [2]. BAC typically arises in the periphery of the lung and grows along alveolar walls, without destruction of the underlying parenchyma and without vascular and pleural invasion [3]. It is characterized by unique epidemiology, clinical features, radiological presentation and cytological characteristics. BAC has long intrigued physicians and oncologists. As BAC becomes a more recognized entity within the pathological continuum of adenocarcinoma, several controversies have emerged regarding this tumor. Herein, we present a new observation of a bronchioloalveolar carcinoma.

## Patient and observation

**Figure 1:**
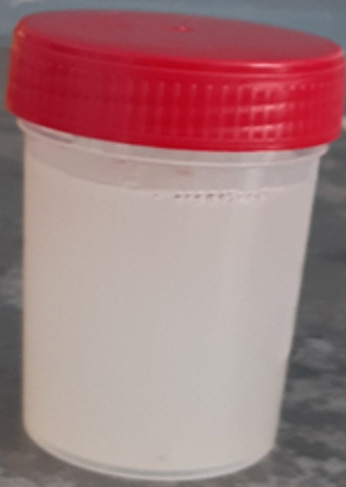
Sample of our patient’s abundant bronchorrhea

**Figure 2:**
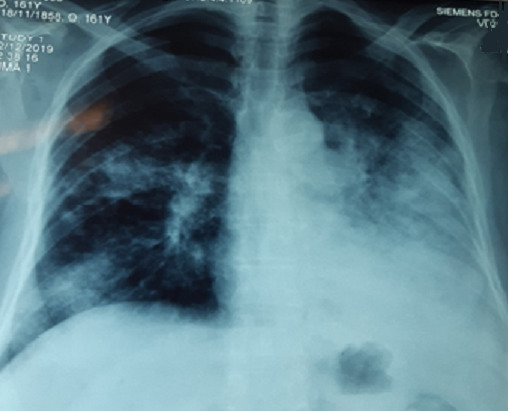
The posteroanterior chest roentgenogram showing a heterogeneous opacity occupying the lower half of the left thoracic hemichamps associated with heterogeneous nodular opacities confluent on the right

**Figure 3:**
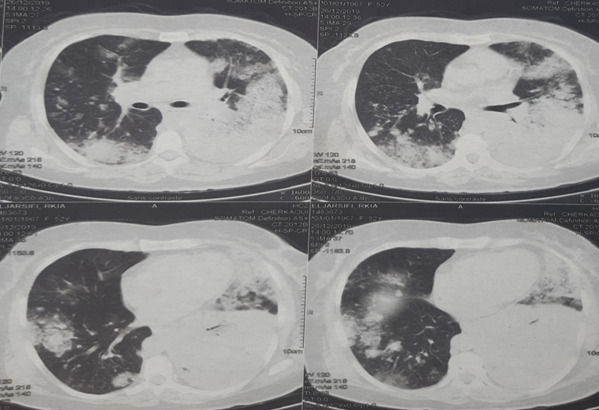
The thoracic computed tomography (CT) showing an alveolar condensation of the left lower lobe containing an air bronchogram, associated with multiple nodular lesions and alveolar condensation of the right lung

**Figure 4:**
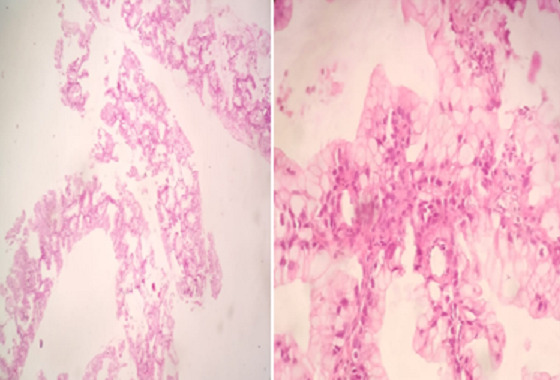
Anatomopathological aspect of invasive mucinous adenocarcinoma lepidic in our patient

Mrs. JR a 50-year-old woman, never treated for tuberculosis and no notion of smoking, but has been diabetic for 5 years. The patient reported for 9 months, a persistent cough with mucous expectoration and very abundant bronchorrhea (1000ml/day) (Figure 1) and stage III of mMRCdyspnea, in a context of apyrexia and deterioration of the general state. Clinical examination revealed a bilateral crackling rale, more marked on the left. The posteroanterior chest roentgenogram showed a heterogeneous opacity occupying the lower half of the left thoracic hemichamps associated with heterogeneous nodular opacities confluent on the right (Figure 2). The thoracic computed tomography (CT) showed an alveolar condensation of the left lower lobe containing an air bronchogram, associated with multiple nodular lesions and alveolar condensation of the right lung (Figure 3). Bronchial fibroscopy not supported by the patient due to abundant bronchorrhea. Transthoracic biopsy of the alveolar condensation was performed (guided by CT) by using the biopsy needle Gelman type (18 G 11 cm). It is concluded from invasive mucinous lepidic adenocarcinoma (Figure 4). The extension assessment did not show extrathoracic localization. After the confirmation of the diagnosis, the patient was referred to the oncology center for chemotherapy.

## Discussion

Lung cancer is the leading cause of cancer mortality worldwide, 85% of cases being non-small cell lung cancers (NSCLC) [4]. The most frequent NSCLC type is invasive lung adenocarcinoma (LUAD). In 2011, a multidisciplinary committee eliminated the term bronchioloalveolar cell carcinoma and divided pulmonary adenocarcinomas into five types: adenocarcinoma in situ, minimally invasive adenocarcinoma, lepidic predominant nonmucinous adenocarcinoma, invasive mucinous adenocarcinoma and invasive adenocarcinoma and its subtypes. The types that would most likely be correlated with an alveolar filling appearance pathologically and on chest imaging are invasive mucinous adenocarcinoma, in which consolidation and air-bronchograms may be present and lepidic-predominant nonmucinous adenocarcinoma, in which a ground-glass appearance is characteristic. Both types of adenocarcinoma are among those formally characterized as bronchioloalveolar cell carcinoma [5].

CBA is a rare tumor that represented only about 5% of the 11,969 consecutive NSCLC in an American epidemiological series [6]. The increase in its incidence suggested in recent years remains debated. However, nearly 30 to 50% of peripheral pulmonary adenocarcinoma contains a non-mucinous type CBA component and the proportion of CBA is much higher in the Asian epidemiological series [2]. CBA is associated with certain epidemiological characteristics that distinguish them from other subtypes of adenocarcinome, but which remain controversial [2] such as an earlier age of onset and a greater female preponderance. The higher proportion of non-smokers (30 to 50%) or light smokers among patients with CBA has however been confirmed by certain case-control studies [2], which perfectly matches the case of our new observation. These epidemiological characteristics are also clearer for the populations of the countries of Southeast Asia.

Pneumonic, multiple and diffuse nodular forms are most often symptomatic [2]. Symptoms are non-specific. It is nevertheless necessary to point out the evocative character, although rather rare of bronchorrhea [2]. It is a mucous expectoration which can be very abundant (up to 500 ml/day), as with our patient and sometimes responsible for progressive respiratory failure. The physical examination may find crackle groans on auscultation and rarely digital hippocratism (‹‹ 10%). Unexpectedly, the general condition of these patients is frequently preserved [7]. Radiographic patterns vary and can include localized disease with peripheral solitary or multiple nodules or masses in 60% of cases or a persistent pneumonic pattern in 40% of cases. The radiographic findings of consolidation with air bronchograms are often initially thought to be consistent with acute pneumonia, but the typical clinical presentation is that of a nonresolving peripheral density on chest radiograph.

In addition, CT may show areas of ground-glass attenuation. Positron emission tomography may be normal because of the low glucose uptake of these tumors. The diagnosis of invasive mucinous adenocarcinoma and lepidic predominant nonmucinous adenocarcinoma is most often made by bronchoscopy with transbronchial biopsy. For staging and treatment, these types of adenocarcinoma are approached like other types of non-small cell lung cancers [7]. Testing for epidermal growth factor receptor (EGFR) mutations should be performed and chemotherapy planned accordingly. In general, the invasive mucinous adenocarcinomas are KRAS positive and EGFR negative. The lepidic predominant nonmucinous type tends to be EGFR positive. Bilateral lung transplantation has been performed, but recurrence in the transplanted lungs has been reported. Tumor progression is essentially intrapulmonary, then bilateral [2, 8].

The low frequency of pleural and mediastinal lymph node extension, the predominance of metastatic bone rather than cerebral localizations and the rarity of hepatic and adrenal metastases [2] should also be emphasized. In the epidemiological series of Zell *et al.* of 11,969 consecutive NSCLCs the presence of a CBA component was an independent criterion for better survival (HR = 1.71) [6]. In addition, the median survival for all stages of CBA and ADC-CBA was greater than 53 months compared to 10 months for the other NSCLCs. Finally, it remained significantly higher for the different TNM stages (I-IIIA: 98 vs 47 months; IIIB: 47 vs 16 months and IV: 10 vs 5 months, p ‹‹ 0.0001). Regardless of the TNM stage, certain characteristics of CBA and ADC-CBA that are easy to collect for some of them at the time of diagnosis constitute prognostic factors for survival. The female sex and the absence of smoking seem to have a favorable prognostic role [2, 6].

Tumors with multiple nodule-like presentation have a better prognosis than those with single or multifocal pneumonic presentation, and diffuse (Tx) [2, 8]. The presence of bronchorrhea or crackles in pulmonary auscultation would have an unfavorable prognosis [2, 9]. Within stages IIIB, it would be necessary to distinguish tumors classified T4 by multiple attack within the same lobe which have a better prognosis than tumors with pleural or N3 lymph node involvement [6]. Similarly, tumors classified M1 by pulmonary involvement affecting more than one lobe have a better prognosis than patients classified M1 due to an extrathoracic metastasis [6], moreover, patients with multiple lobe involvement within the same lung (unilateral involvement) have a better prognosis than patients with both lung involvement (bilateral involvement) [6].

## Conclusion

Bronchioloalveolar carcinoma (BAC) is a rare subtype of lung adenocarcinoma, the feature of our observation, compared to the literature, is the Similarity of the age of onset, of the female sex and of the typical clinical, radiological and histological characteristics. Through our work we insist on thinking of lepidic mucinous carcinoma in the face of any persistent alveolar opacity.

## Competing interests

The authors declare no competing interests.
